# Exploring the Relationship Between Eye Movements and Electrocardiogram Interpretation Accuracy

**DOI:** 10.1038/srep38227

**Published:** 2016-12-05

**Authors:** Alan Davies, Gavin Brown, Markel Vigo, Simon Harper, Laura Horseman, Bruno Splendiani, Elspeth Hill, Caroline Jay

**Affiliations:** 1 Department of Computer Science, University of Manchester, Manchester, United Kingdom; 2 Department of Medicine, University of Sheffield, Sheffield, United Kingdom; 3 Department of Library and Information Science, University of Barcelona, Barcelona, Spain; 4 Department of Surgery, Washington University, Saint Louis, United States

## Abstract

Interpretation of electrocardiograms (ECGs) is a complex task involving visual inspection. This paper aims to improve understanding of how practitioners perceive ECGs, and determine whether visual behaviour can indicate differences in interpretation accuracy. A group of healthcare practitioners (n = 31) who interpret ECGs as part of their clinical role were shown 11 commonly encountered ECGs on a computer screen. The participants’ eye movement data were recorded as they viewed the ECGs and attempted interpretation. The Jensen-Shannon distance was computed for the distance between two Markov chains, constructed from the transition matrices (visual shifts from and to ECG leads) of the correct and incorrect interpretation groups for each ECG. A permutation test was then used to compare this distance against 10,000 randomly shuffled groups made up of the same participants. The results demonstrated a statistically significant (α  0.05) result in 5 of the 11 stimuli demonstrating that the gaze shift between the ECG leads is different between the groups making correct and incorrect interpretations and therefore a factor in interpretation accuracy. The results shed further light on the relationship between visual behaviour and ECG interpretation accuracy, providing information that can be used to improve both human and automated interpretation approaches.

The electrocardiogram, commonly referred to as the EKG or ECG, is a graphical representation of the electrical activity generated by heart cells that is detectable on the body surface. This is made possible by amplifying and filtering waves of electrical current generated by cardiac cells via electrodes placed on the surface of the body^1^. The ECG is a widely used clinical test that is both affordable and easily repeatable^2^,^3^. Apart from detection of cardiac pathology, the ECG is also used to detect a wide variety of other conditions, including metabolic disturbances, such as hyperkalaemia, the presence of artificially implanted cardiac devices (e.g. pacemakers, AICDs) and toxic levels of certain drugs^1^,^2^,^3^. Correct interpretation of the ECG is vital for clinical decision making leading to appropriate subsequent treatment of patients^3^,^4^,^5^. ECG interpretation can be a difficult task. Even expert cardiologists viewing the same ECG can disagree significantly on many aspects of interpretation^6^. Failure to interpret potentially fatal ECG findings has led to significant sums (millions of dollars) being spent on litigious processes and malpractice payouts, in addition to having a significant impact on the quality of patients’ lives^4^. Increasing demand is being placed on physicians to interpret ECGs without access to complete clinical information, especially regarding the triage of emergency patients suspected of having an acute myocardial infarction. Physicians are often asked to review or interpret ECGs from people with suspected myocardial infarctions (MI) accessed via telemedicine systems in this way in order to streamline emergency cardiac care^7^. Viewing ECGs without firsthand clinical information can lead to false MI diagnosis and increased costs due to activation of cardiac catheterization labs. In some cases patients undergo unnecessary angiography with no subsequent arterial occlusion^7^. Despite the estimated 300,000,000 ECGs that are recorded every year^8^, there is still a paucity of standardized guidelines for ECG interpretation, with different hospitals, educational facilities and other institutions all using their own protocols to teach and assess competency of ECG interpretation^9^. It is also the case that individuals sometimes develop their own interpretation techniques and may lack awareness of their own limitations, falsely believing that they interpret ECGs well^3^. Although computational ECG interpretation continues to improve it has still not reached a level where it can be relied upon with confidence^4^. Computer-derived ECG interpretations are often erroneous and frequently require human expert over-reading^10^. Computerized interpretation accuracy is further reduced in the presence of arrhythmias^6^. Several studies have also identified significant discrepancies in computerized interpretation when obtained a few minutes apart from the same stable patients, casting doubt over the tenability of such interpretation approaches for clinical decision making^6^.

The investigation of differences between the cognitive processes that take place within individuals who carry out this complex task accurately, as opposed to those who do not, may offer a means of improving both human and computer interpretation. One method that has the potential to provide such insights is eye-tracking^5^. Analyzing visual behavior using eye-tracking technology has been applied to various medical domains, most notably the field of radiology where performance relating to nodule detection on radiographs was improved when practitioners could see the areas of the image others had searched, regardless of whether they were viewing expert or novice gaze patterns^11^. Krupinski noticed an increased fixation duration and count of fixations on image areas containing the abnormality, even if the practitioner did not recognize the significance. Superimposing this location over the image was also shown to improve performance^12^. More recently, eye-tracking technology has been applied specifically to the domain of ECG interpretation to gain a better understanding of perceptual skill in this area^5^,^9^,^13^,^14^,^15^. One study has shown that ECG leads that are viewed first are also viewed for the longest time, with the largest fixation duration in lead V1 and the shortest in V4 across 12 different ECGs^9^. Another study, involving students taking an electrocardiography module on a healthcare science degree, has demonstrated a negative correlation between the time taken to reach an interpretation, and performance. In this case, participants fixated the rhythm strip the longest and lead I the least^13^. Wood *et al*. compared the visual behavior and performance of expert and novice groups with and without a brief patient history. They discovered that interpretation accuracy was related to the time taken to fixate leads displaying salient features of the condition in question, and that experts were faster to fixate these leads. Presence of clinical history was not found to influence the way the image was observed^14^. These previous studies all highlight links between eye-movements and visual search tasks, indicating that visual behaviour between individuals does differ depending on the task, especially between expert and novice groups.

Previous studies have considered accuracy in the context of allocating participants *a priori* to expert and novice groups^14^,^15^ or calculating the overall percentage accuracy across a number of stimuli (ECGs)^9^. This is the first study to group participants by accuracy for each stimulus. This is an important distinction as individuals may make correct and incorrect interpretations interchangeably when viewing different stimuli. The study reported here focuses on 12-lead resting ECGs for human adults, with the primary aim of adding to the growing knowledge base encompassing visual perception related to ECG interpretation. In particular, it proposes a method for analyzing visual transitions between ECG leads as a function of interpretation accuracy. There is wide consensus among ECG teaching texts that clues to the presence of certain abnormalities are best found by viewing specific leads, depending on the abnormality in question^1^. This is specifically relevant in STEMI cases, where the involvement of specific lead territories is used to determine the area of the heart that is affected and help distinguish the STEMI from other conditions that also present with ST-segment elevation (Table 1).

Therefore the cross referencing of certain leads may be of more or less importance depending on the abnormality in question. We hypothesize: 1) that there would be higher frequencies of visual transitions from and to the key lead areas associated with the condition in question, and other salient features unique to the stimulus, in the group of participants that made the correct interpretation when compared to the group who did not; 2) that overall there would be a measurable difference in the visual transition behavior between the group of participants that made the correct interpretation, and the group who made an incorrect interpretation.

To explore the impact of visual transitions between leads we use a distance metric to determine how different the transition patterns are for the groups of people making correct and incorrect interpretations compared with randomly mixed groups. A permutation test is carried out to generate a sampling distribution and associated p-value (described in detail in methods).

## Results

### Participant performance

**Figure 1:**
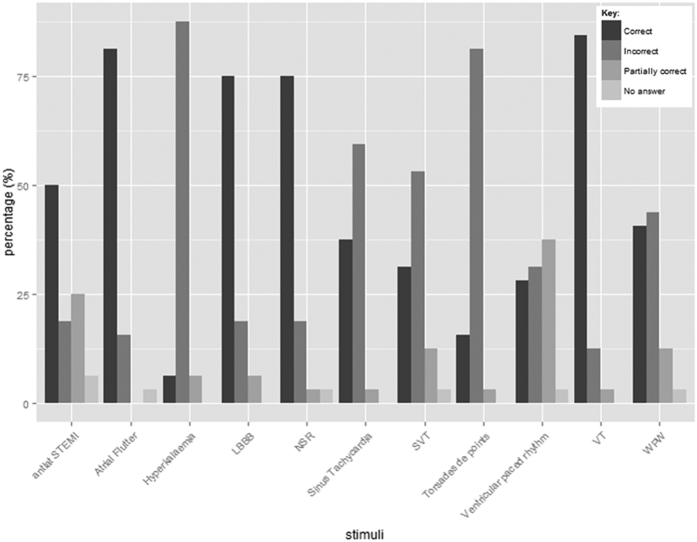
Interpretation accuracy per stimulus.

**Figure 2:**
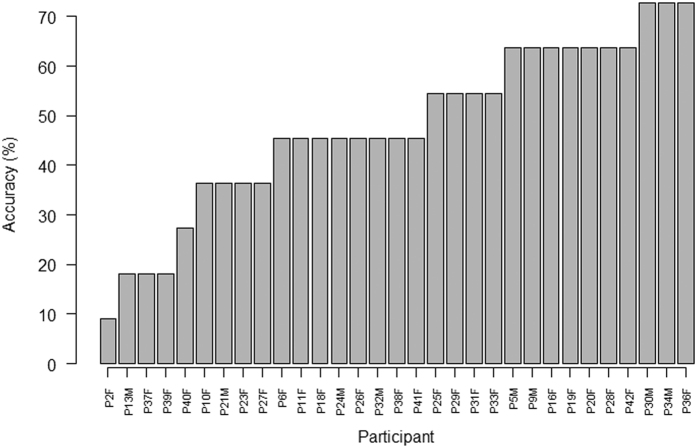
Percentage accuracy per participant.

The performance of the participants varied widely across the stimuli presented (Fig. 1). Participants performed poorly in identifying the hyperkalaemia (6.3% correct) and torsades de pointes (15.6% correct) stimuli. In comparison VT and atrial flutter were identified correctly by 84% and 81% of participants respectively. The percentage of correct interpretations made by individuals (Fig. 2) shows that accuracy ranges from 9% to 73%. The top three participants scoring above 70% were a consultant cardiac physiologist (CP), advanced CP and CP. The lowest scoring participant was a third year medical student, who identified 9.1% of conditions correctly. Over half of participants (n = 17) had an interpretation accuracy of below 50%. None of the participants was able to identify all the stimuli correctly, with the best among them making incorrect interpretations in over a quarter of cases.

An independent 2-group Mann-Whitney U Test was carried out on the aggregated group of students 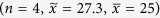 and the remaining professionals 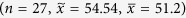. The test showed that there was a statistically significant difference between the accuracy levels of the group, demonstrating that professionals were more accurate in their interpretations than the students (*W* = 9, *p* < .01).

### Fixation duration and count

**Figure 3:**
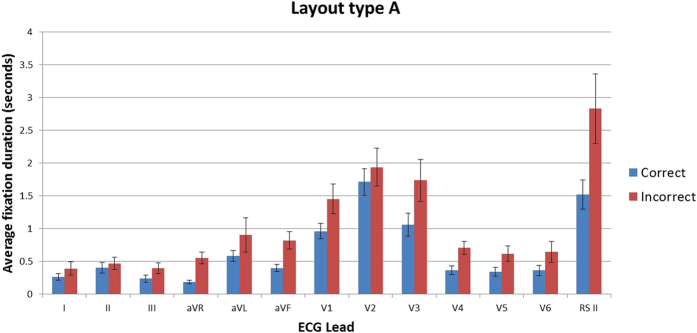
Mean fixation duration in seconds (all stimuli all leads) layout type A.

**Figure 4:**
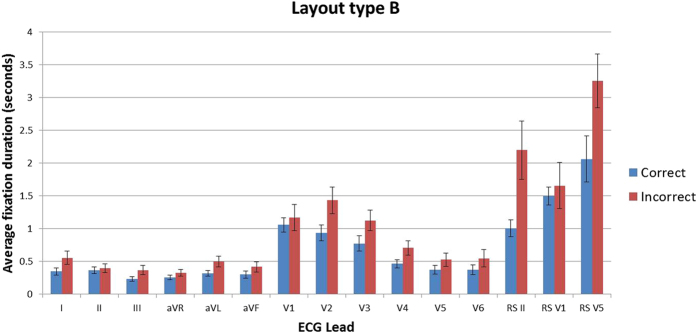
Mean fixation duration in seconds (all stimuli all leads) layout type B.

**Figure 5:**
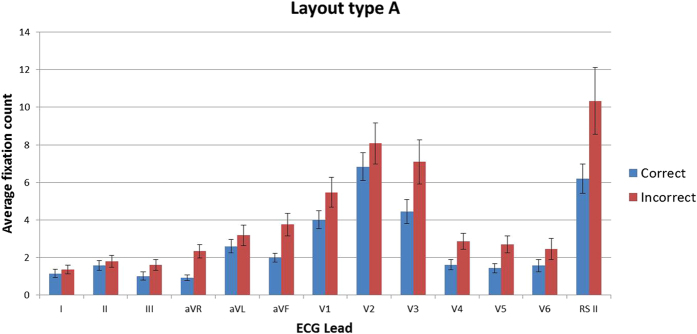
Mean fixation count (all stimuli all leads) layout type A.

**Figure 6:**
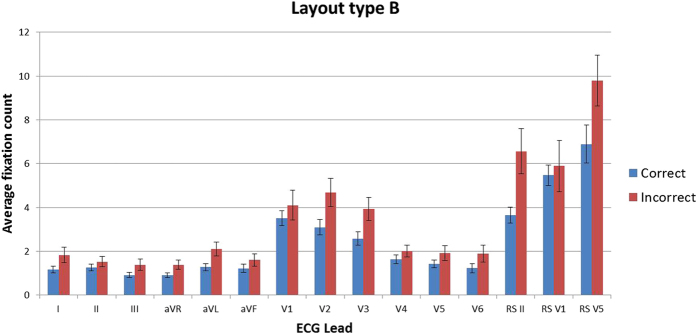
Mean fixation count (all stimuli all leads) layout type B.

Descriptive statistics show that in all leads for both layout types across all stimuli, the mean fixation duration and the mean fixation count are lower in the correct group than the incorrect group, indicating that practitioners who make incorrect interpretations make more fixations on each lead, and that individual fixations are longer. On stimuli of layout type A, the incorrect group made the longest fixations on rhythm strip II (3.68 s) whereas the correct group made them on lead V2 (1.83 s). In stimuli with layout type B the lead with the greatest fixation durations for both groups was rhythm strip V5 (correct = 2.28 s, incorrect = 3.55 s) (Figs 3 and 4). The fixation count is the number of fixations made in each lead. The average fixation count for leads in stimuli with layout type A that were fixated the most often for both groups were, lead V2 for the correct group (7.3 fixations) and rhythm strip II for the incorrect group (13.4 fixations). The least fixated lead was aVR for the correct group (1.7 fixations) and lead I for the incorrect group (2.7 fixations). Rhythm strip V5 in layout type B was fixated the most times by both groups (correct = 7.6, incorrect = 10.6 fixations) with lead aVR being the least fixated lead by both groups (correct = 1.7, incorrect = 2.3 fixations) (Figs 5 and 6). This highlights a potential global difference in visual behavior between the two groups based on the duration of fixations and the fixation count. The results also suggest that the incorrect group focuses more on the rhythm strips than the correct group does.

### Scan paths

**Figure 7:**
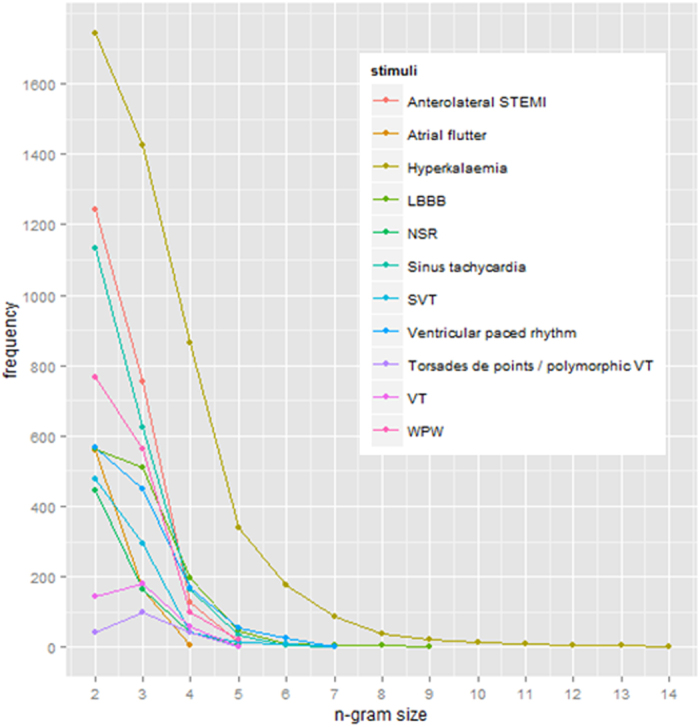
N-gram size and frequency per stimulus.

N-gram analysis was used to analyze the sequence of AOIs visited, also known as a scan path. Scan path sequences were represented as strings of letters representing the AOIs. Figure 7 shows the size of the n-gram and its frequency of occurrence, and demonstrates that shorter sequences occur with higher frequency. In all stimuli apart from Hyperkalaemia (n-gram length = 14) and LBBB (length = 9) the length is below 7, with the highest frequencies occurring between n-gram lengths of 2 and 4. An n-gram length of 2 was found with the highest frequency in 9 of the 11 stimuli. As bi-gram sequences occur most frequently in the majority of stimuli, a first order Markov process was considered suitable to capture the visual transitions from and to various leads on the ECGs.

### Jensen-Shannon distance

**Figure 8:**
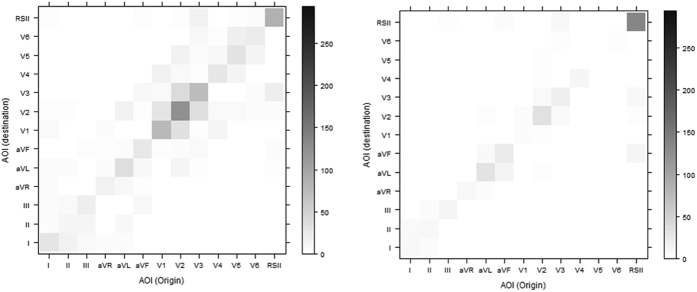
Transition matrices for the anterolateral STEMI groups: left correct, right incorrect.

The transition matrices (Fig. 8) that are constructed as an intermediate stage in the JSD calculation show some differences in transition behavior between the two groups. Figure 8 shows the transition matrices of the correct/incorrect groups for the Anterolateral STEMI stimulus. The darker the square the higher the number of transitions. The correct group has a higher frequency of transitions occurring in and between the precordial leads V1-V4, where the ST-elevation is at its greatest (>5 mm in V2 and V3). ST-elevation is the main salient feature in all ST-elevation myocardial infarctions (STEMIs)^1^.

Transition matrices provide insight into visual transition behavior from a descriptive perspective. To determine whether the observed differences in the visual transition patterns of the correct and incorrect groups were statistically significant, the JSD calculation was applied with a permutation test.

**Figure 9:**
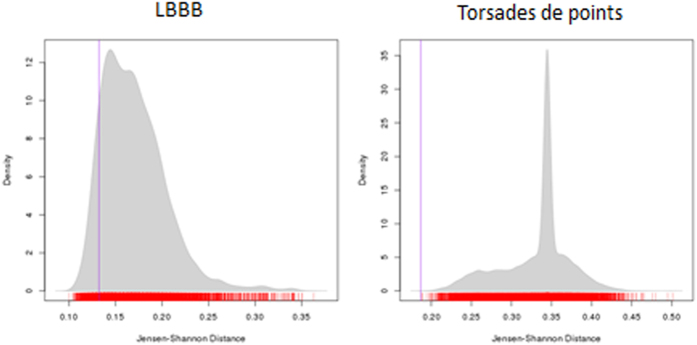
JS distance permutation distributions *α*  0.05 (vertical line represents correct/incorrect distance).

**Figure 10:**
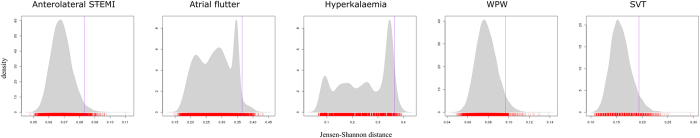
JS distance permutation distributions (vertical line represents correct/incorrect distance).

**Figure 11:**
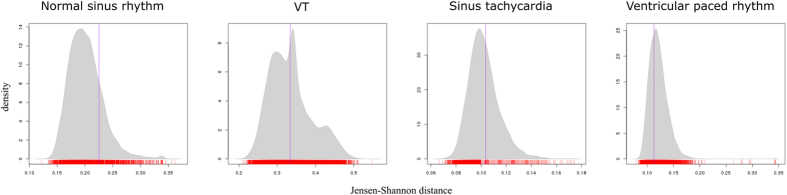
JS distance permutation distributions (vertical line represents correct/incorrect distance).

There was a statistically significant difference between the two groups (*α*  0.05) for five of the stimuli: Anterolateral STEMI, Atrial Flutter, Hyperkalaemia, SVT and WPW (Table 2 and Fig. 9). In all five cases the distance between the correct/incorrect groups is significantly larger than between randomly mixed groups, indicating that the visual transitions between leads on these ECGs is a factor in the accuracy of their interpretation. A further four stimuli (Normal sinus rhythm, VT, sinus tachycardia and ventricular paced rhythm) showed no statistically significant difference between groups, suggesting that transitions made no more difference to accuracy than random chance (Fig. 10). The final two stimuli – LBBB (*p* = 0.89) and torsades de pointes (*p* = 0.99) – demonstrated the opposite, showing that the distance between the groups was significantly smaller than those generated randomly (Table 2, Fig. 11). This indicates that some factor other than accuracy was influencing the behaviour of the groups on these stimuli.

## Discussion

This work contributes to our understanding of ECG interpretation, by proposing a technique to quantify differences in gaze behaviour as a function of interpretation accuracy. We expand on previous work by 1) going beyond the use of standard eye-tracking metrics, such as fixation duration/count, to consider the cross-referencing of different regions of the ECG, and 2) grouping participants on a *post hoc* basis according to the accuracy of their interpretation, rather than their level of experience or job role. We discovered that there was a statistically significant difference between the visual transition patterns of groups making correct and incorrect interpretations on five of the eleven ECGs examined, demonstrating that for certain cardiac pathologies, the way in which the ECG is viewed may be important for accurate interpretation. There is the potential to utilize such knowledge to improve training and/or assessment of expertise. Accurate interpretation of the ECG is a challenging task, even for experts. Bond *et al*.^9^ recruited 21 expert annotators during the International Society of Computerised Electrocardiology (ISCE) conference in 2013, with an average of over thirty years’ experience interpreting ECGs. The experts scored a mean accuracy of 63% compared with the 48% mean score of participants in this study. These results suggest that even with greater experience, ECG interpretation is still challenging. Traditional methods of teaching ECG interpretation often involve practitioners examining each part of the ECG waveform (the various waves, intervals and segments) for morphological variations from the norm^13^. In contrast, experts are known to use schemes based around the structure of organized knowledge used for diagnostic reasoning^16^. Previous work has been undertaken looking at the differences between expert and novice groups^14^ although as indicated in this study and others^9^,^17^, even experts/specialists make mistakes. This led us to look specifically at the differences between those making correct and incorrect interpretations, as opposed to the broader categories of experts and novices.

The link between cognition and eye movement patterns has been increasingly widely accepted within the field of visual cognition research^18^,^19^. Many eye tracking metrics, such as fixation duration, fixation count and time to first fixation are proxies of visual behavior and can be used as a coarse means of describing the phenomena in question. Attempting to model the visual search task applied to complex stimuli such as ECGs requires an approach that also captures domain expertise. One of the challenges that exists with these proxies relates to the context and the task, meaning that many of the standard eye tracking metrics commonly used, such as fixation duration, time to first fixation and fixation count will vary greatly depending on the specific task being studied. An example can be seen with the fixation duration, where longer fixation durations can be interpreted as a sign of expertise in activities such as chess and art. However longer fixations in vigilance research can indicate shallow processing or near daydreaming, for example drivers traveling along information-poor roads^19^.

Many ECG texts and training courses make reference to viewing certain leads to best observe the salient features of a specific condition (for example^1^,^20^). These specific leads vary from condition to condition and are only present in certain ECG manifestations. Other conditions present with salient features that can be seen in various lead locations and are not necessarily best viewed in particular leads. With ECGs where correct interpretation is incumbent on known cross-referencing requirements, such as STEMIs, one would expect individuals making correct interpretations in these instances to cross-reference the relevant leads to determine the sub type of STEMI. This phenomenon was noted by Wood *et al*.^14^ who observed the scan path of an expert and novice viewing an inferior STEMI. It appeared that the expert was cross-referencing the ST-elevation in the inferior leads with the precordial leads. The current study also provides a quantitative demonstration of this phenomenon, in the transition matrix of the aggregated group of people who made a correct interpretation for the anterolateral STEMI stimulus.

Using the Jensen-Shannon distance calculation with a permutation test allowed us to measure the similarity between the two sets of probability distributions of the Markov chains, and thus compare the difference between the correct and incorrect groups with the difference between randomly constructed alternative groups. Permutation testing allows computational power to be utilized when sufficient data is not otherwise available to construct a model of events and the data is non-parametric^21^. This technique can also be useful in compensating for smaller sample sizes.

Previous work has demonstrated differences between expert and novice groups’ visual behavior in the domain of ECG interpretation. Wood *et al*.^14^ found that experts were twice as fast to locate the critical leads on the stimuli presented, although no differences in the search rate was identified between the two groups. Wood *et al*.^14^ describe critical leads as those leads containing the most salient information about the condition.

The greater confidence and faster time for fixation of experts identified by Wood *et al*.^14^ could go some way toward explaining why the correct group had a lower fixation duration and count than the incorrect group. This may be because the correct group were faster and more confident at identifying and extracting meaningful information from their visual inspection of the different leads. The incorrect group requires more time to identify the features seen in these areas. As searching for a target within a scene is predominately a reactive process in most tasks^22^, it may be that both experts and novices begin viewing the ECG in this way, with experts then able to understand the significance of an identified salient feature and apply pattern recognition strategies to the task. Pattern recognition is often used by clinical experts when interpreting medical images. Rapid information retrieval, triggered by salient clues, allows experienced practitioners to match up what they are seeing with similar cases they recall from the past^16^. This potentially complex cognitive process is not usually available to less experienced novice practitioners^16^, who are left to search the ECG exhaustively, with an inefficient systematic approach^13^. Correct diagnostic reasoning has been identified as being more likely to be achieved using pattern recognition strategies than other reasoning techniques, such as hypothetico-deductive reasoning^16^. Expertise in accurately interpreting ECGs are also thought to be attributable to superior pattern-recognition skills^14^. This has been found to be a factor in radiograph interpretation, with pattern recognition established as a performance indicator for correct interpretation^23^. Although pattern recognition can be difficult for beginners, and memorising a pattern without understanding it is not ideal^24^, it is still considered to be a core element of ECG interpretation^2^. Repeated exposure in the order of a minimum of 500 ECGs (seen under expert supervision) is considered to meet the standard of competence in interpretation^2^.

The fact that visual transitions can play a greater or lesser role in correct interpretation may explain why there is a significant difference between groups in 5 of the stimuli but no difference in 4 of them. As the anterolateral STEMI is a type of condition that can only be correctly identified by the presence of the salient feature (ST elevation) in specific leads, it would be expected that transitions would be important to accurately identifying the condition. Arguably the inferior leads and rhythm strip are good places to examine to identify flutter waves associated with atrial flutter^20^. The Wolff-Parkinson White syndrome (WPW) is identified by a short PR interval and an up sloping delta wave that can be present in multiple leads, although experts can also identify the location of the accessory pathway and distinguish between WPW types A and B by looking at the defection of the QRS complex in leads V1 and V2^1^,^20^. Conditions such as Hyperkalaemia, VT, normal sinus rhythm and the ventricular paced rhythm present their own salient features in multiple leads and for accurate diagnosis it is not necessary to look in certain pre-defined areas. It is therefore not clear why visual transition behaviour is different between accurate and inaccurate groups for the Hyperkalaemia stimulus, but it is interesting to note this effect, as it may reveal that there are still ‘better’ ways to view the ECG, even though it is technically possible to diagnose the condition from any lead. The LBBB and torsades de pointes stimuli seem to show that the transition patterns of the correct and incorrect groups are almost identical. It would appear that in these cases factors other than visual transitions are at work. This systematic similarity could be an effect of training, or alternatively the two groups could be attracted to the same salient features but using different prior knowledge in the case of the correct group to infer meaning.

The technique described provides a way of determining the presence and significance of the effect gaze shift has on the accuracy of ECG interpretation. This has the potential to be used for training, where the gaze patterns of people making correct diagnoses could be shown explicitly, or correspondence between a trainee’s and expert’s gaze patterns could be compared. The technique could also potentially be used to stratify ECG conditions into those that rely on these predetermined leads, and those that do not. There is also potential for this knowledge to feed into automated ECG interpretation approaches, by showing how, for example, cross-referencing of leads may aid the diagnostic process.

The study has some methodological limitations. The differing layouts and variety of conditions made direct comparisons between stimuli challenging. Participants viewing the stimuli on a computer screen in a quiet room and being allowed unlimited time to arrive at an interpretation is less ecologically valid than the busy hospital environment, where distractions are present and time is often limited. Additionally the sizes of the correct and incorrect groups differed greatly in many of the stimuli. This may have added a degree of artifact to the results of some stimuli where the difference in group size was especially large. Nevertheless, the sample size here is comparable to that of other eye-tracking studies in this area. The relatively small number of participants is counterbalanced by the richness and depth of data obtained.

This exploratory study focused on first order Markov chains, constructed from transition bi-grams. It is possible that n-gram extraction of the AOIs visited could be modeled with higher order Markov chains to examine the more complex sequence of visual transitions across an entire stimulus in order. Combining this with temporal data may help to reveal the presence of more complex relationships between fixation time/duration and visual transition sequence.

The analysis technique presented here is useful because it provides a means of identifying conditions where gaze shift is pertinent to making a correct interpretation. It also provides a means of quantifying the difference between correct and incorrect interpretation groups. There are several implications of this work, including highlighting the potential for eye-tracking to be used for the assessment of expertise and for training purposes, by identifying conditions where accuracy is more or less influenced by visual transitions. A deeper understanding of the differences between practitioners who make accurate interpretations and those who do not can help to improve aspects of ECG interpretation, and ultimately enable more reliable clinical decisions to be made.

This study also has potential implications for both training and decision making. Participants often fixated on the same salient features regardless of their accuracy, indicating that people may be subconsciously detecting anomalies, and that ECG viewing may be enhanced through ‘perceptual feedback’: highlighting on the image areas where pracitioners have looked the most, to encourage them to consciously consider these areas in more detail. Perceptual feedback has been shown to improve accuracy in detecting pulmonary nodules in radiography^11^,^25^, and these results show that applying it to ECG interpretation could also improve accuracy and/or human training in the field of electrocardiology.

## Methods

### Equipment

Tobii 1750 and Tobii X2-60 eye trackers were used to capture eye tracking data. Additional equipment used included: a 17” monitor; Tobii Studio 3.2 software; a keyboard and mouse; a Zoom H2 Handy Recorder and microphone for audio recording.

### Procedure

Forty-three participants from medical/healthcare backgrounds took part in the study. Eighteen different 12-lead ECGs obtained from on-line open access libraries (www.lifeinthefastlane.com/ecg-library and www.emedu.org/ecg_lib/index.htm) were displayed in random order on a computer screen, using Tobii studio software. Each participant could view each ECG for as long as required before moving onto the next. Participants spoke their interpretation of the ECG aloud and moved through the stimuli by clicking a mouse button. Two additional ECGs were used for pre-test calibration checks. The participants’ eye tracking data was recorded as they viewed the ECGs and attempted interpretation. Informed consent was obtained for all participants and ethical approval provided by the University of Manchester, school of Computer Science Research Ethics Comity (CS65). All methods were performed in accordance with the relevant guidelines and regulations.

Participants with a recording quality below 70% (the threshold recommended by the manufacturer, Tobii) were not included in the analysis. Recording quality pertains to a percentage derived from the number of eye tracking samples that the software identifies divided by the number of attempts. Looking away from the screen or the software not being able to identify one or both eyes reduces the recording quality percentage.

This left 31 participants, predominantly from a cardiac physiology background (Tables 3 and 4). Participants had an average of 9.3 and median of 6 years experience experience interpreting ECGs (Table 3). The study was exploratory, and the ECGs were chosen to represent a variety of conditions. As this study is looking specifically at accuracy, ECGs that were potentially ambiguous were excluded from the analysis. This paper presents the results of 11 of the stimuli. This subset was chosen as in every case there was no ambiguity regarding what the correct interpretation should be, and they presented clear examples of the morphology required to identify the underlying medical conditions they represented.

**Figure 12:**
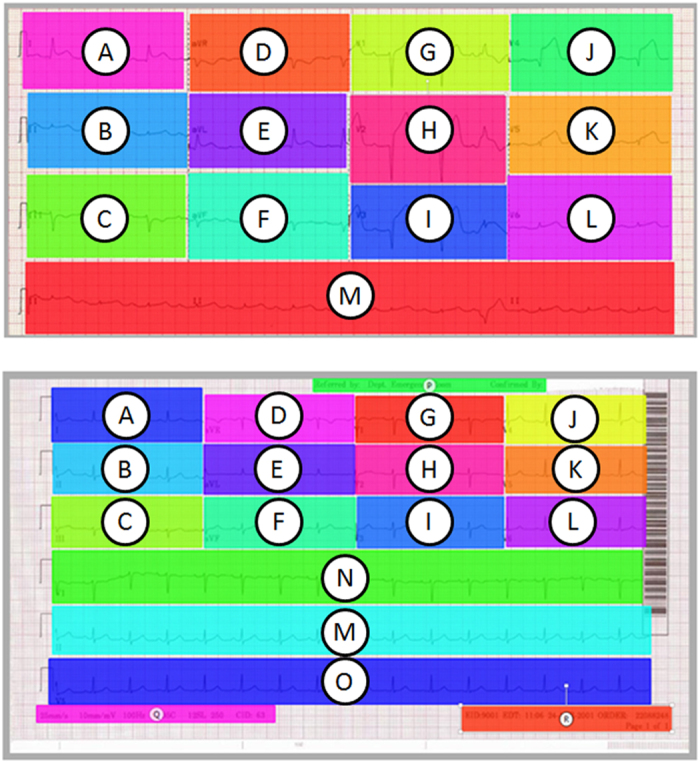
Layout types. Top: (**A**) bottom (**B**) Equation 1. Jensen-Shannon Distance.

Tobii eye tracking software was used to divide the 12-lead ECGs into different areas of interest (AOIs). AOIs were created for each of the standard ECG leads (I, II, III, aVR, aVL, aVF and V1 - V6). Additionally AOIs were created for the rhythm strip(s) displayed underneath the standard leads and any additional textual annotations shown on the ECGs. The stimuli varied in the number of additional rhythm strips displayed on the ECG. Five of the stimuli had a single rhythm strip (lead II) displayed beneath the standard 12 leads, the other 6 stimuli displayed 3 rhythm strips (V1, II and V5). These stimuli were classified as layout type A or layout type B respectively (Table 5). Layout type A ECGs had AOIs labeled A-M. Type B AOIs were labeled A-R (Fig. 12).

The Tobii I-VT filter was used with its default settings, and minimum fixation duration was set to the default 60 ms, as fixations of this duration are often observed during reading^26^,^27^. As the ECG is a graph that also contains text, lead names and textual annotations, the use of this lower fixation duration threshold was considered appropriate.

Six of the ECGs chosen had key leads that literature describes as the best place to usually see the salient features of the specific condition; the remaining five featured conditions that were not associated with changes documented as being related to specific leads (Table 6).

Two people classified the interpretations made as either correct or incorrect. The classifiers consisted of the lead author of this paper who intepreted ECGs in clinical settings and authored several texts on ECG interpretation. The second individual was an experienced independent ECG annotator (doctoral research fellow in emergency medicine whose research focuses on ECG interpretation). There was a 1.8% (6/341 cases) difference in agreement which was resolved through discussion. Correct answers would include the precise name of the condition in question, rather than just describing some of the salient features. Common variations in expression of the same condition were also accepted as correct, i.e. normal sinus rhythm, sinus rhythm and NSR would all be accepted as correct. Partially correct answers would include answers that described the main features of the condition without specifically naming it. Examples include saying bundle branch block, instead of stating specifically if it was a left or right bundle branch block, or saying STEMI or MI instead of anterolateral STEMI. Additionally, answers were marked as partially correct if they included the correct answer but also described other conditions along with the correct answer that were not present on that stimulus. Answers were marked as incorrect if they stated a completely different interpretation of the condition such as atrial fibrillation instead of LBBB.

Previous research has explored differences between experts and novices when interpreting ECGs^14^,^15^. This paper looks explicitly at visual behavior as a function of *diagnostic accuracy*, from the perspective of whether the gaze patterns of practitioners who make correct interpretations consistently differ from those who do not. Although this approach has arguably more ecological validity, the resulting data is generally not suitable for standard inferential analysis procedures. Group sizes (correct/partially correct/incorrect) tend to be unequal, and vary greatly between stimuli. For the purpose of analysis the partially correct was amalgamated into the incorrect group leaving us with two groups for analysis (correct and incorrect). This decision was taken to ensure the categorisation process was robust and repeatable: whilst it is relatively straightforward to identify whether an interpretation matches a correct diagnosis, it is much harder, with a partially correct interpretation, to decide whether it is mostly right, or mostly wrong, and therefore to determine which category to put it in. For this reason, we decided to delineate clearly between completely correct, and all other answers. There are also significant variations in within-participant accuracy, as the same individual may make correct and incorrect interpretations across the different stimuli. The ECG literature often suggests cross-referencing the different salient features of the various conditions^1^, meaning we also cannot assume independence of the ECG leads. Identifying a salient feature in a certain lead may cause the practitioner to check different subsequent leads than they may otherwise have done. This relationship between the various different waveform morphologies in the leads is key to building the overall picture of the condition presented. This study looks specifically at the visual transitions made between one lead and another in the context of interpretation accuracy.

In order to better understand the differences between the two groups at the stimulus level and provide a quantifiable comparison, a computational technique was used. The raw data files for each stimulus were exported from Tobii studio for analysis. Bespoke program scripts were created using the R project for statistical computing. The scripts were used to extract fixation events corresponding to hits in the AOIs for the lead sections of the ECGs from both layout types. Unclassified gaze events and saccades were excluded. Transition matrices of the aggregated frequency of transitions from and to the various leads of the ECG for the correct and incorrect groups were constructed respectively. The transition matrices were then converted into first-order Markov chains, using the maximum likelihood estimates of the transition probabilities. A Bayesian prior was added to each cell of the matrices to remove the potential issue of division by zero errors in the computation. The Jensen-Shannon divergence (based on the Kullback-Leibler (KL) divergence) was then computed between the two Markov chains. This divergence is commonly used to provide a measure of similarity between two probability distributions. The square root of the divergence was calculated to derive the Jensen-Shannon Distance metric (JSD) (Equation 1).


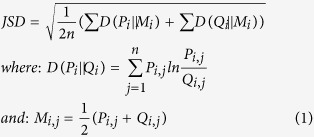


This metric satisfies the properties of a distance measure, such as fulfillment of the triangle inequality^28^ and is a true metric, as opposed to a pseudo-metric, which makes it suitable for use as a distance measure between the two Markov chains. The analysis process was repeated for randomly partitioned groups of the same size (containing both correct and incorrect interpretation participants). A permutation test^21^ was carried out 10,000 times to estimate the sampling distribution of an unknown statistic. A p-value can then be calculated as a fraction of the permutation values greater than the original non-permuted value^21^. If there is something “special” about the correct group when compared to the incorrect group then we would expect the distance between them to be larger than between two groups chosen at random. A significance level of 5% was used for all statistical tests. We hypothesize that visual transitions will have a greater impact on accuracy where there are multiple key leads that require cross-referencing (i.e. anterolateral STEMI), as opposed to ECGs where the condition can be identified by fixating the majority of leads or any single lead.

## Additional Information

**How to cite this article**: Davies, A. *et al*. Exploring the Relationship Between Eye Movements and Electrocardiogram Interpretation Accuracy. *Sci. Rep.* **6**, 38227; doi: 10.1038/srep38227 (2016).

**Publisher's note:** Springer Nature remains neutral with regard to jurisdictional claims in published maps and institutional affiliations.

## Figures and Tables

**Table 1:** STEMI lead regions related to cardiac anatomical orientation.

STEMI territories	Myocardial area	Possible artery involved
II, III, aVF	Inferior	Right coronary
I, aVL, V5, V6	Lateral	Circumflex
V1, V2, V3, V4	Anterior	Left anterior descending

**Table 2:** Jensen-Shannon distance results per stimulus * = *p*  0.05.

Stimuli	JS distance	p-value	Group 1 (n)	Group 2 (n)
Anterolateral STEMI*	0.2884499	0.02	16	15
Atrial Flutter*	0.6041648	0.03	25	6
Hyperkalaemia*	0.6038147	0.03	2	29
LBBB	0.3634885	0.90	23	8
Normal Sinus Rhythm	0.4745519	0.20	23	8
Sinus Tachycardia	0.3218995	0.40	12	19
Supraventricular Tachycardia*	0.4400723	0.05	10	21
torsades de pointes	0.4320926	1.00	5	26
Ventricular paced rhythm	0.3360781	0.70	9	22
Ventricular Tachycardia	0.5778351	0.50	26	5
Wolff-Parkinson-White syndrome*	0.3110491	0.04	12	19

**Table 3:** Study participants.

Participant clinical role	n	%
Cardiac physiologist/technician	20	62.5
Student cardiac physiologist/technician	4	12.5
Registrar	1	3.1
Cardiac registrar	2	6.3
Medical student	1	3.1
Senior nurse	1	3.1
Consultant	1	3.1
FY2 doctor	1	3.1
Health care assistant	1	3.1
**Gender**		
Male	8	26
Female	23	74

**Table 4:** Participant background and experience level.

Participant	Background	ECG interpretation experience
P2F	3rd year medical student	Short period of intensive training + text book learning
P5M	Cardiology physiologist	4 years experience post training
P6F	Senior nurse	2.5 years experience post training
P9M	Cardiology registrar	Not available
P10F	Cardiology technician	6.5 years experience since training
P11F	Cardiology technician	Not available
P13M	Student cardiac physiologist	2nd year
P16F	Cardiology technician	20 years
P18F	Cardiology technician	10 years
P19F	Healthcare Assistant	2 years
P20F	Cardiac physiologist	15 years
P21M	Cardiac physiologist	4 years
P23F	Student cardiac physiologist	2nd year
P24M	Cardiology registrar	4 years
P25F	Cardiac physiologist	2 years
P26F	Advanced cardiac physiologist	20 years
P27F	Student cardiac physiologist	2nd year
P28F	Cardiac physiologist	2 years
P29F	Cardiac physiologist	1 year
P30M	Consultant cardiac physiologist	20 years
P31F	Advanced cardiac physiologist	25 years
P32M	Registrar	8 years
P33F	Consultant	22 years
P34M	Advanced cardiac physiologist	15 years
P36F	Cardiac physiologist	6 years
P37F	Ex-cardiac physiologist	7 years
P38F	Advanced cardiac physiologist	30 years
P39F	Cardiac physiologist	6 years
P40F	Cardiac physiologist	18 years
P41F	Cardiac physiologist	3 years
P42F	FY2 Doctor	2 years, non-specific

**Table 5:** Stimuli layout types.

Layout type A	Layout type B
Anterolateral STEMI	Left bundle branch block (LBBB)
Atrial flutter	Normal sinus rhythm (NSR)
Hyperkalaemia	Sinus tachycardia
torsades de pointes/polymorphic ventricular tachycardia	Supraventricular tachycardia (SVT)
Wolff-Parkinson-White syndrome (WPW)	Ventricular paced rhythm
	Ventricular tachycardia (VT)

**Table 6:** Key leads by ECG.

Stimuli	Key leads
Anterolateral STEMI	I, aVL, V1 - V6
Atrial Flutter	Rhythm strip
Hyperkalaemia	None
LBBB	V1, V6
Normal Sinus Rhythm	None
Sinus Tachycardia	Rhythm strip
Supraventricular Tachycardia	Rhythm strip
torsades de pointes	None
Ventricular paced rhythm	None
Ventricular Tachycardia	None
Wolff-Parkinson White syndrome	V1-V3

## References

[b1] Wagner G. Marriott’s Practical Electrocardiography 11^th^ edn (Lippincott Williams & Wilkins, 2008).

[b2] Kadish a. H. . ACC/AHA clinical competence statement on electrocardiography and ambulatory electrocardiography: A report of the ACC/AHA/ACP-ASIM task force on clinical competence (ACC/AHA Committee to develop a clinical competence statement on electrocardiography and am. Circulation 104, 3169–78 (2001). 11748119

[b3] Eslava D., Dhillon S., Berger J., Homel P. & Bergmann S. Interpretation of electrocardiograms by first-year residents: the need for change. Journal of electrocardiology 42, 693–7 (2009). 19740482 10.1016/j.jelectrocard.2009.07.020

[b4] Mele P. Improving electrocardiogram interpretation in the clinical setting. Journal of electrocardiology 41, 438–9 (2008). 18572183 10.1016/j.jelectrocard.2008.04.003

[b5] Bond R. R. . Eye Tracking in the Assessment of Electrocardiogram Interpretation Techniques. Computing in Cardiology 581–584 (2012).

[b6] Salerno S. M., Alguire P. C. & Waxman H. S. Competency in Interpretation of 12-Lead Electrocardiograms: A Summary and Appraisal of Published Evidence. Annals of Internal Medicine 138, 751–760 (2003). 12729431 10.7326/0003-4819-138-9-200305060-00013

[b7] McCabe J. M. . Physician accuracy in interpreting potential ST-segment elevation myocardial infarction electrocardiograms. Journal of the American Heart Association 2, e000268 (2013). 24096575 10.1161/JAHA.113.000268 PMC3835230

[b8] Holst H., Ohlsson M., Peterson C. & Edenbrandt L. A confident decision support system for interpreting electrocardiograms. Clinical Physiology 19, 410–418 (1999). 10516892 10.1046/j.1365-2281.1999.00195.x

[b9] Bond R. R. . Assessing computerized eye tracking technology for gaining insight into expert interpretation of the 12-lead electrocardiogram: an objective quantitative approach. Journal of electrocardiology 47, 895–906 (2014). 25110276 10.1016/j.jelectrocard.2014.07.011

[b10] Anh D., Krishnan S. & Bogun F. Accuracy of electrocardiogram interpretation by cardiologists in the setting of incorrect computer analysis. Journal of electrocardiology 39, 343–5 (2006). 16777525 10.1016/j.jelectrocard.2006.02.002

[b11] Litchfield D., Ball L. J., Donovan T., Manning D. J. & Crawford T. Learning from others: effects of viewing another person’s eye movements while searching for chest nodules. Medical Imaging 6917, 691715–691715 (2008).

[b12] Krupinski E. A., Calvin N. F. & L H. K. Enhancing recognition of lesions in radiographic images using perceptual feedback. Optical Engineering 37, 813–818 (2013).

[b13] Breen C. J., Bond R. & Finlay D. An evaluation of eye tracking technology in the assessment of 12 lead electrocardiography interpretation. Journal of electrocardiology 47, 922–9 (2014). 25200901 10.1016/j.jelectrocard.2014.08.008

[b14] Wood G., Batt J., Appelboam A., Harris A. & Wilson M. R. Exploring the Impact of Expertise, Clinical History, and Visual Search on Electrocardiogram Interpretation. Medical Decision Making 34, 75–83 (2014). 23811761 10.1177/0272989X13492016

[b15] Broadbent M., Horsley M., Birks M. & Persaud N. Comparing Novice and Expert Nurses in Analysing Electrocardiographs (ECGs) Containing Critical Diagnostic Information: An Eye Tracking Study of the Development of Complex Nursing Visual Cognitive Skills. In Horsley M., Eliot M., Knight B. A. & Reilly R. (eds.) Current Trends in Eye Tracking Research, chap. 24, 133 (Springer-Verlag, London, 2014).

[b16] Coderre S., Mandin H., Harasym P. H. & Fick G. H. Diagnostic reasoning strategies and diagnostic success. Medical education 37, 695–703 (2003). 12895249 10.1046/j.1365-2923.2003.01577.x

[b17] Novotny T. . Data analysis of diagnostic accuracies in 12-lead electrocardiogram interpretation by junior medical fellows. Journal of Electrocardiology 48, 988–994 (2015). 26381796 10.1016/j.jelectrocard.2015.08.023

[b18] Thomas L. E. & Lleras A. Moving eyes and moving thought: on the spatial compatibility between eye movements and cognition. Psychonomic bulletin & review 14, 663–8 (2007). 17972730 10.3758/bf03196818

[b19] Holmqvist K. . Eye tracking: A comprehensive guide to methods and measures (Oxford University Press, New York, 2011).

[b20] Jenkins R. D. & Gerred S. J. ECGs By Example 2^nd^ edn, (Elsevier, 2005).

[b21] Knijnenburg T. a., Wessels L. F. a., Reinders M. J. T. & Shmulevich I. Fewer permutations, more accurate P-values. Bioinformatics 25, 161–168 (2009). 10.1093/bioinformatics/btp211 PMC2687965 19477983

[b22] Nicolaou M., James A., Darzi A. & Yang G.-z. A Study of Saccade Transition for Attention Segregation and Task Strategy in Laparoscopic Surgery. In Medical Image Computing and Computer-Assisted Intervention â€“ MICCAI 2004, 97–104 (Berlin, 2004).

[b23] Krupinski E. a. & Berbaum K. S. The Medical Image Perception Society Update on Key Issues for Image Perception Research1 253, 230–233 (2009). 10.1148/radiol.2531090237 PMC2817829 19709995

[b24] Willis J. Special Article Methods Used to Interpret the 12-Lead Electrocardiogram : Pattern Memorization versus the Use of Vector Concepts 3 (2000). 10.1002/clc.4960230103 PMC6655172 10680023

[b25] Krupinski E. a. Enhancing recognition of lesions in radiographic images using perceptual feedback. Optical Engineering 37, 813 (1998).

[b26] Salojärvi J. . Inferring Relevance from Eye Movements : Feature Extraction. Workshop at NIPS 2005, In Whistler, Canada on December 1-, 2005 (2005).

[b27] Radach R., Huestegge L. & Reilly R. The role of global top-down factors in local eye-movement control in reading. Psychological research 72, 675–88 (2008). 18936964 10.1007/s00426-008-0173-3

[b28] Endres D. & Schindelin J. A new metric for probability distributions. Information Theory, IEEE. 49, 1858–1860 (2003).

